# Editorial Brevities

**Published:** 1904-01

**Authors:** 


### EDITORIAL BREVITIES.

DR. FRANK H. SIMS has removed his office to the English-American Building.

DR. A. G. THOMAS, a well-known eclectic physician and professor in the Eclectic Medical College, died recently.

DR. J. MC. F. GASTON, JR., was one of the winners in the *International Journal of Surgery's* prize contest.

DR. H. M. PAYNE, the father of Dr. Clarence Payne, a well-known homeopathic physician of Atlanta, died recently of pneumonia. He had at one time been president of the American Institute of Homeopathy.

### A GASTRONOMIC TRAGEDY.

A gluttonous Frenchman hippophagous,
Wedged a chunk of horse meat in th' esophagus;
Though they pushed and they shoved
It couldn't be moved;
And now he rests in his sarcophagus.

THE regular meeting of the Atlanta Society of Medicine was held on December 17. After the usual programme, the annual election of officers was held. The officers for the year are: Dr. A. W. Sterling, President; Dr. E. Bates Block, Vice-President; Dr. W. B. Emery, Secretary; Dr. E. Van Goidtsnoven, Treasurer; Dr. L. B. Clarke, member of Executive Committee.

Dr. Sterling's popularity, ability and professional zeal will insure for the society a year of progress and prosperity.

